# Case Report: An Unusual Course of Angiosarcoma After Lung Transplantation

**DOI:** 10.3389/fimmu.2021.789851

**Published:** 2022-01-03

**Authors:** Saskia Bos, Liesbeth Daniëls, Lucienne Michaux, Isabelle Vanden Bempt, Sascha Vermeer, FJ Sherida H Woei-A-Jin, Patrick Schöffski, Birgit Weynand, Raf Sciot, Sabine Declercq, Laurens J. Ceulemans, Laurent Godinas, Geert M. Verleden, Dirk E. Van Raemdonck, Lieven J. Dupont, Robin Vos, the Leuven Lung Transplant Group, Jonas Yserbyt

**Affiliations:** ^1^ Department of Respiratory Diseases, University Hospitals Leuven, Leuven, Belgium; ^2^ Translational and Clinical Research Institute, Newcastle University, Newcastle upon Tyne, United Kingdom; ^3^ Histocompatibility and Immunogenetics Laboratory (HILA), Red Cross-Flanders, Mechelen, Belgium; ^4^ Center for Human Genetics, University Hospitals Leuven, Leuven, Belgium; ^5^ Department of General Medical Oncology, University Hospitals Leuven, Leuven Cancer Institute, KU Leuven, Leuven, Belgium; ^6^ Department of Pathology, University Hospitals Leuven, Leuven, Belgium; ^7^ Department of Pathology, ZNA Middelheim Hospital, Antwerp, Belgium; ^8^ Department of Thoracic Surgery, University Hospitals Leuven, Leuven, Belgium; ^9^ Department of CHROMETA, Laboratory of Respiratory Diseases and Thoracic Surgery (BREATHE), KU Leuven, Leuven, Belgium

**Keywords:** donor-related, lung transplantation, malignancy, case report, angiosarcoma

## Abstract

A 35-year-old woman underwent bilateral lung transplantation for primary ciliary dyskinesia and developed vascular tumors over a slow time course. Initial presentation of non-specific vascular tumors in the lungs and liver for up to 6 years after transplantation evolved toward bilateral ovarian angiosarcoma. Tumor analysis by haplotyping and human leukocyte antigen typing showed mixed donor chimerism, proving donor origin of the tumoral lesions. In retrospect, the donor became brain dead following neurosurgical complications for a previously biopsy-proven cerebral hemangioma, which is believed to have been a precursor lesion of the vascular malignancy in the recipient. Donor-transmitted tumors should always be suspected in solid organ transplant recipients in case of uncommon disease course or histology, and proper tissue-based diagnosis using sensitive techniques should be pursued.

## Introduction

Malignancy is a known complication after solid organ transplantation related to the use of immunosuppression and early development of age-associated neoplasms (1). The prevalence of malignancies (including skin cancer and post-transplant lymphoproliferative disease) at 5 and 10 years after lung transplantation is 24.6% and 44.2%, respectively (2). In contrast, donor-related malignancies are rare (3, 4).

## Case Description

A 35-year-old woman underwent bilateral lung transplantation for primary ciliary dyskinesia in 2013. The donor was a 44-year-old female who became brain dead following neurosurgical complications for a previously biopsy-proven cerebral hemangioma. Chest and abdominal CT, performed prior to organ allocation, excluded concurrent vascular lesions or metastases. The lungs, both kidneys, and liver were allocated for transplantation to four different recipients. Our recipient was treated with tacrolimus, mycophenolate, and steroids as standard maintenance immunosuppressive regimen.

**Figure 1:**
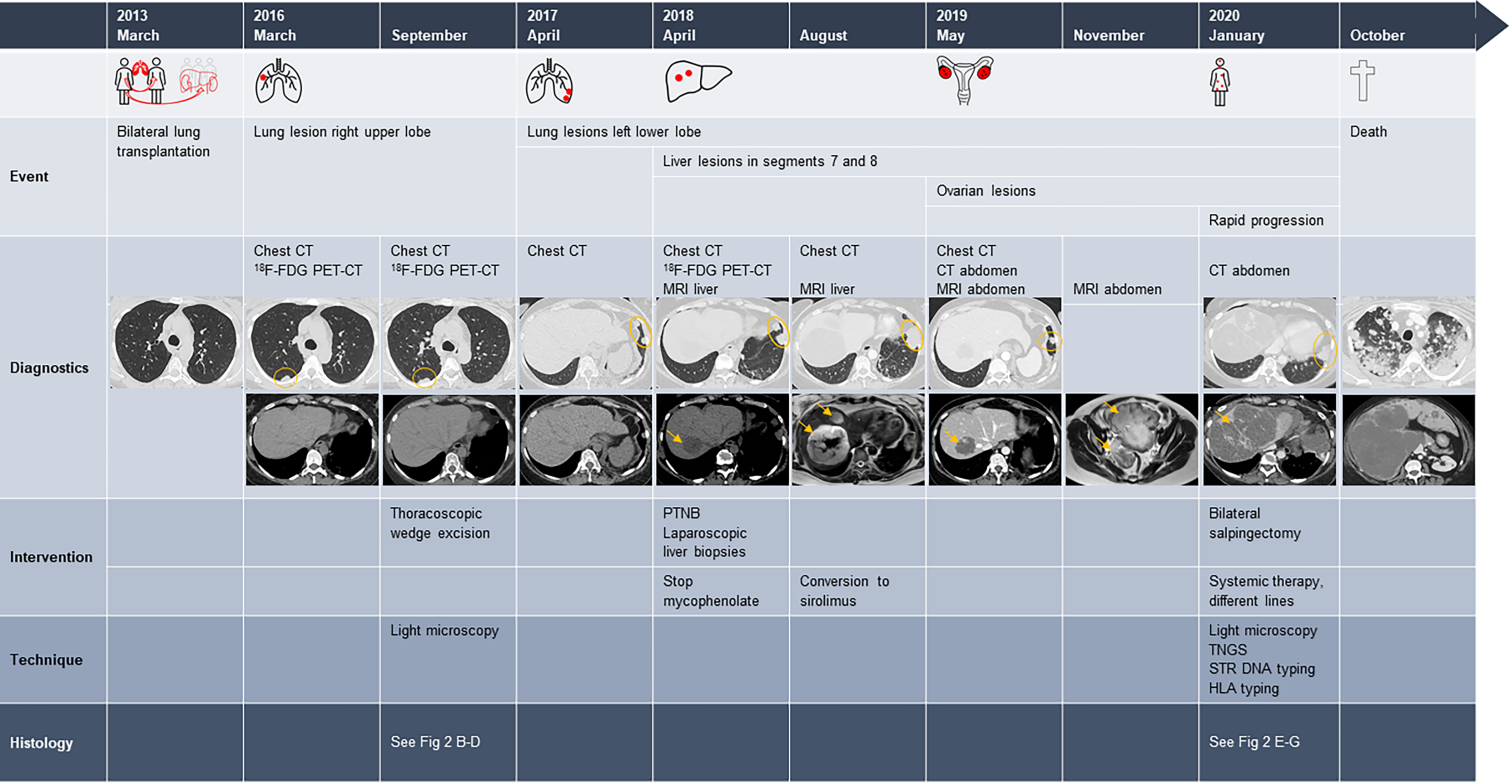
Time course of events after lung transplantation. ^18^F-FDG PET-CT, ^18^F-fluorodeoxyglucose PET-CT; PTNB, percutaneous transthoracic needle lung biopsies. Radiography images: 1) chest CT of March 2013, axial reconstruction pulmonary window: normal lung parenchyma. 2) Chest CT of March and September 2016, axial reconstruction pulmonary window: nodule in the upper right lobe. Axial reconstruction soft tissue window: normal liver parenchyma. 3) Chest CT of April 2017, axial reconstruction pulmonary window: two nodules at the left costodiaphragmatic sinus. Axial reconstruction soft tissue window: normal liver parenchyma. 4) Chest CT of April 2018, axial reconstruction pulmonary window: two nodules at the left costodiaphragmatic sinus; axial reconstruction soft tissue window: largest of two hypodense liver lesions with a diameter of 38 mm. 5) Chest CT of August 2018, axial reconstruction pulmonary window: two lung nodules have merged into one large lesion. Hepatic MRI, T2-weighted axial reconstruction: partly real increase in volume and partly due to intralesional bleeding. 6) Chest and abdominal CT of May 2019, axial reconstruction pulmonary and soft tissue window: significant decrease in volume of the lung and liver lesions after conversion to sirolimus. 7) MRI abdomen of November 2019, T2-weighted axial reconstruction: enlarged ovaries, especially the left one, with mixed solid tissue lesions. 8) CT abdomen of January 2020, axial reconstruction pulmonary window: volume increase of the lung lesion at the left costodiaphragmatic sinus; axial reconstruction soft tissue window: massive tumor progression of the liver lesions. 9) Chest and abdominal CT of October 2019, axial reconstruction pulmonary and soft tissue window: massive tumor progression of the lung and liver lesions. Large figures of the radiography can be found in the **Supplementary Materials**.

In 2016, a 15-mm poly-lobular nodule was detected in the right upper lobe on routine chest CT (**Figure 1**, March 2016) without increased metabolism on ^18^F-fluorodeoxyglucose PET-CT (^18^F-FDG PET-CT), and further follow-up was provided. A slight increase to 20 mm was detected 6 months later, but again ^18^F-FDG PET-CT revealed no FDG avidity (**Figure 1**, September 2016). Because of the increase in volume, thoracoscopic wedge excision was performed. Histological examination revealed several fragments of thickened fibrous pleura and emphysematous alveolar parenchyma, without signs of malignancy.

Follow-up CT 6 months later demonstrated two small nodules of 11 and 15 mm in the costodiaphragmatic sinus of the left lower lobe (**Figure 1**, April 2017), for which a conservative approach was adopted. However, a gradual volume increase was observed after 6 and 12 months, and two additional hypodense liver lesions appeared, the largest with a diameter of 38 mm (**Figure 1**, April 2018). ^18^F-FDG PET-CT again demonstrated no local hypermetabolism in either the lung or liver lesions. Hepatic MRI showed T2-intermediate to hyperintense lesions, with intralesional and peripheral contrast enhancement, yet no precise diagnosis could be made. Due to a lack of clarity about histology and evolution of the lesions over time, mycophenolate was discontinued. To obtain accurate tissue diagnosis, a transthoracic biopsy of the most dorsal lung lesion was performed, but histology was non-diagnostic. Histological examination of laparoscopic liver biopsies showed liver parenchyma with fragments of aberrant vascular proliferation, characterized by irregular vascular channels and delineated by large cells with irregularly defined nuclei, but without noticeable hyperchromasia. Focal apoptotic debris and one mitotic figure were present. The lesions were positive for CD31 and CD34 (endothelial cell markers) and α-smooth muscle actin (an actin isoform that predominates within vascular smooth muscle cells, in which its expression is often less or absent in malignancies). The conclusion was undetermined vascular lesions without signs of malignancy, but with an uncertain biological behavior. The lung wedge excision was revised given this information, and one vascular lesion, consistent with a hemangioma, was noted.

**Figure 2:**
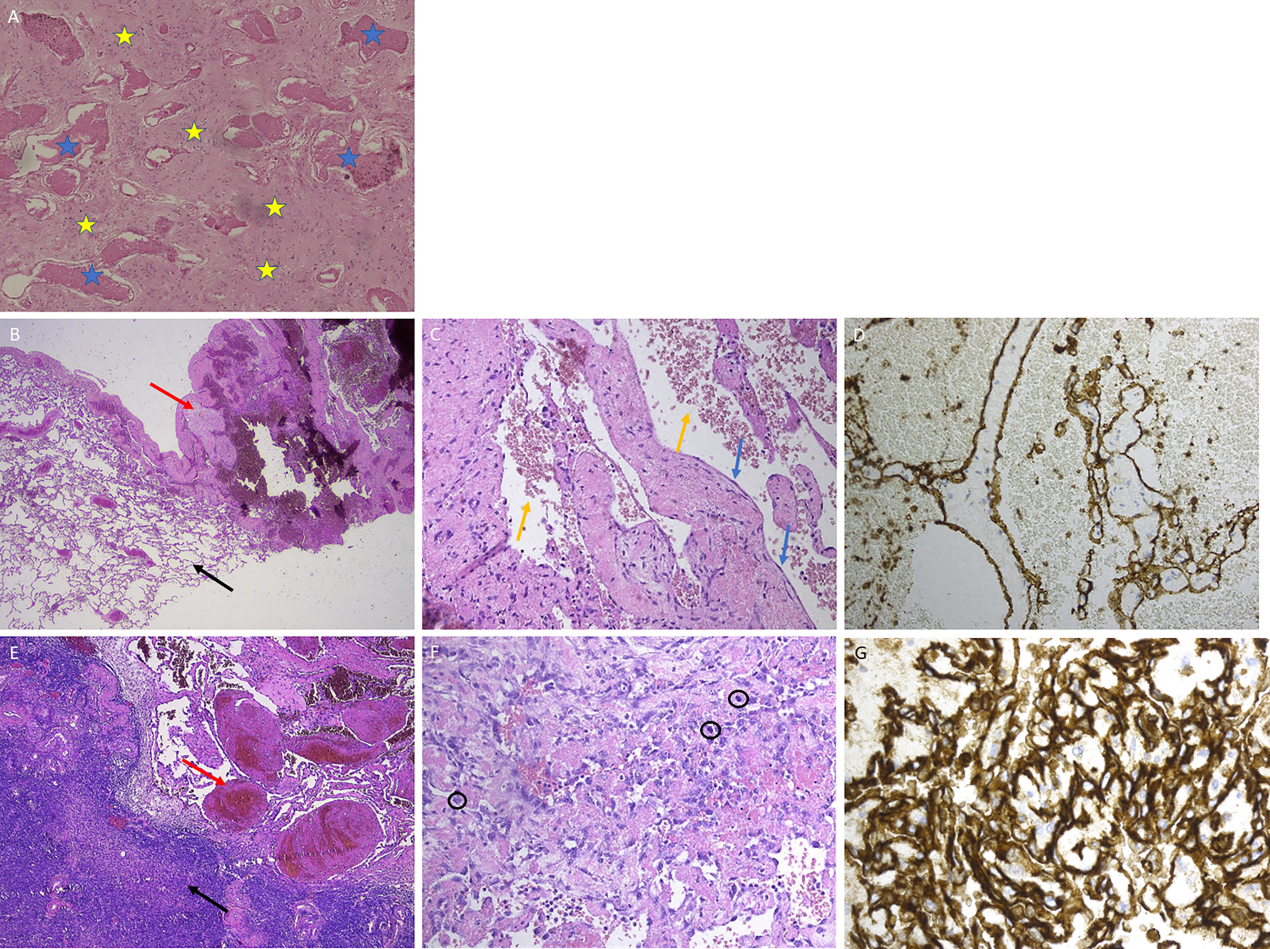
Histology images of donor and recipient lesions. **(A)** Histology image of donor brain lesion: irregular thin-walled vascular spaces, delineated by a flattened endothelium (blue asterisk). The vessels are embedded in gliotic tissue (yellow asterisk). **(B–D)** Lung lesion (recipient, September 2016). **(B)** Low power view of the lung lesion, showing the transition from the lung tissue (left, black arrow) to the vascular lesion (right, red arrow). **(C)** Dilated blood-filled spaces (yellow arrows), delineated by a normal flat endothelium (blue arrows). **(D)** CD31 stain (brown) confirms that the spaces are lined by endothelium. **(E–G)** Ovarian lesion (recipient, January 2020). **(E)** Transition from ovary cortical tissue (left, black arrow) to vascular lesion (right, red arrow). **(F)** Irregular vascular spaces delineated by hyperchromatic, atypical endothelial cells with numerous mitoses (circles). **(G)** CD31 stain (brown) shows the lesion’s chaotic architecture.

MRI of the liver 4 months later showed a significant increase of both lesions, due to a combination of real volume increase and spontaneous hemorrhage. However, a concurrent further increase of the lung lesions was noted (**Figure 1**, August 2018). Based on available data, benign or low-grade malignant vascular malformations were suspected, and tacrolimus was changed to sirolimus because of its anti-proliferative effects on vascular smooth muscle and endothelial cells (5–7). Consecutive CT scans showed a significant volume decrease of the lung and liver lesions over the next 9 months (**Figure 1**, May 2019). However, at this stage, both ovaries had increased significantly in size. MRI demonstrated mixed solid tissue lesions with a preference for benign vascular lesions, but re-evaluation 6 months later revealed a further volume increase (**Figure 1**, November 2019). Bilateral salpingectomy was performed, and histological examination established a definitive diagnosis of bilateral ovarian *angiosarcoma*. Microscopically, the tumors were consistent with vascular lesions with clear focal atypia, piling of nuclei, and excessive mitotic figures; were positive for vascular markers (CD31 and CD34); and had a local absence of α-smooth muscle actin (**Figure 2**). Targeted next-generation sequencing (TNGS) analysis showed identical gene variants in the ovarian and lung lesions, highly suggestive of hematogenous metastatic spread from the lung to ovaries (“Krukenberg” tumor) (**Table 1**).

**Table 1:** TNGS, STR, and HLA analysis.

			Ovarian lesion	Lung lesion
**TNGS**	***Gene*_exon**	**Variant allelic frequency**	%	%
	**Pathogenic variants**			
	*CTNNB1*_exon3	c.133T>C (p.(Ser45Pro))	21	10
	*PIK3CA*_exon9	c.1638G>T (p.(Gln546His))	12	10
	*TERT*_promotor	c.-146C>T (p.)?	33	22
	*CDKN2A*_exon2	c.204_210delinsAG (p.(Pro70Leufs*48))	18	<5
	**Variants of unknown significance**			
	*H3F3A*_ex4	c.362T>G (p.(Met121Arg))	8	6
	*ERBB3*_exon21	c.2569G>C (p.(Asp857His))	11	31
	*GNA11*_exon4	c.548G>A (p.(Arg183His))	11	5
	*APC*_exon16	c.2720G>T (p.(Gly907Val))	19	32
	*FGFR1*_exon12	c.1595T>C (p.(Met532Thr))	21	32
**STR**	**Haploptypes**	**Recipient profile**		
	CSF1PO	10–12	9–10–11–12	
	D13S317	9–11	9–11–13	
	D16S539	9–11	9–10–11	
	D18S51	17	12–17	
	D21S11	31.2–32.2	28–31.2–32.2	
	D3S1358	14–16	14–16–17*–18*	
	D5S818	11–12	11*–12	
	D7S820	8–10	8–10–12	
	D8S1179	14–15	14–15	
	FGA	21–25	21–22–25	
	PENTA_D	9–10	9–10–11	
	PENTA_E	5–17	5–7–17	
	THO1	7–9.3	6–7–9.3	
	TPOX	8–9	8–9–11	
	vWA	17–19	16*–17–19–20*	
**HLA**	**HLA locus**		Donor/recipient	Donor/recipient
	A		A2, A26*/NI	A2, A26*/A2, A3
	B		B38*, B51*/B35, B41	B38*, B51*/B35, B41
	C		NI/NI	NI/NI
	DR		NI/NI	DR103, DR13/DR4, DR13
	DQ		DQ5*, DQ6/DQ6, DQ8	NI/DQ6, DQ8

TNGS, targeted next-generation sequencing; STR, short tandem repeat; HLA, human leukocyte antigen; NI, not interpretable.

TNGS: showing identical gene variants in the left ovarian tumor and lung lesion. TNGS was performed using a custom SeqCap EZ HyperCap hybridization-based capture panel protocol (Roche Sequencing and Life Science Kapa Biosystems, Wilmington, MA) targeting protein coding exons as well as specific targets, including promotor regions or intronic regions (KIT intron 10 and MET intron 13/14) of 96 cancer genes (AKT1, ALK, AMER1, APC, ARAF, ARID1A, ATM, ATRX, BAP1, BCOR, BRAF, BRCA1, BRCA2, CCND1, CCNE1, CDH1, CDK4, CDK6, CDKN2A, CIC, CTNNB1, DDR2, DICER1, EGFR, ERBB2, ERBB3, ERBB4, ERCC2, ESR1, FBXW7, FGFR1, FGFR2, FGFR3, FGFR4, FOXL2, FUBP1, GATA3, GLI1, GLI2, GNA11, GNAQ, GNAS, H3F3A, H3F3B, HIST1H3B, HIST1H3C, HRAS, IDH1, IDH2, KIT, KRAS, LZTR1, MAP2K1, MDM2, MDM4, MET, mTOR, MYC, MYCN, NF1, NF2, NOTCH1, NRAS, PALB2, PDGFRA, PDGFRB, PIK3CA, PIK3R1, POLE, PRDM6, PTCH1, PTEN, RAC1, RAF1, RB1, RET, RICTOR, RNF43, ROS1, SDHA/B/C/D, SMAD4, SMARCA4, SMARCB1, SMO, SPRED1, STK11, SUFU, TERT prom, TP53, TSC1, TSC2, VEGFR3, and WT1). Pooled libraries containing captured DNA fragments were subsequently sequenced on an Illumina NextSeq instrument as 2 × 150 bp paired-end reads with a minimum read depth of at least 500× coverage. Interestingly, the pathogenic CTNNB1 mutation did not result in cytoplasmic or nucleic translocation of β-catenin on immunohistochemistry.

STR: Haplotypes of the ovarian tumor and recipient profiles (with the exclusion of the markers on sex chromosomes), determined by the number of tandem repeats for 15 STR loci using PowerPlex^®^ 16 kit (Promega). * Alleles also found in donor profile (in the donor, only 3 of the 15 markers became interpretable due to insufficient DNA quality, of which D3S1358 and vWA are informative).

HLA, HLA typing of the lung and ovarian lesion, showing the presence of unique donor HLA (marked with *), with ovarian donor HLA A and B identical to donor HLA A and B from the lung lesion, proving donor origin of the ovarian metastases. Not all loci could be analyzed due to insufficient quality of the matter.

Sections of the former lesions were revised, but the conclusions remained unchanged: lung hemangioma with a preserved expression of α-smooth muscle actin, no nuclear atypia, nor mitosis; and vascular liver lesions without signs of malignancy, but with an unclear biological behavior. Similar abnormalities were seen in some areas in the ovaries and were interpreted as potential precursor lesions.

Two weeks postoperatively, because of severe right upper quadrant abdominal pain, massive hepatic tumor progression was observed (**Figure 1**, January 2020), as well as bone metastases, for which systemic treatment with paclitaxel (80 mg/m^2^) was initiated. After eight consecutive weekly administrations, re-evaluation demonstrated disease progression, and systemic treatment was changed to single-agent doxorubicin (75 mg/m^2^) every 3 weeks. Further disease progression was documented after three cycles. Oral treatment with an anti-angiogenic tyrosine kinase inhibitor pazopanib (daily dose of 400 and afterward 800 mg) was initiated. However, 3 months later, further disease progression with lymph node metastases was noted, and fourth-line systemic treatment with gemcitabine (1,000 mg/m^2^ on days 1, 8, and 15 of a 28-day cycle) was started, yet without avail. Best supportive care was initiated upon radiographic development of brain, kidney, and thyroid metastases, and the patient succumbed 2.5 weeks later, 7.6 years after transplantation.

Given the donor’s previous diagnosis of brain hemangioma and the unusual course of multiple, new-onset vascular lesions in the recipient, the question arose whether this could have been a donor-related malignancy. The DNA profile of the recipient and donor was compared, evaluating the number of tandem repeats for 15 short tandem repeat (STR) loci. Briefly, DNA was extracted from formalin-fixed paraffin-embedded tissue of the donor cerebrum and recipient left ovarian tumor using Maxwell^®^ RSC DNA FFPE Kit (Promega), followed by co-amplification and three-color detection of 16 loci (15 STR loci and amelogenin) (PowerPlex^®^ 16 kit, Promega, Madison, Wisconsin, USA). Forty percent of cells of non-recipient origin were present in the ovarian tumor tissue. The DNA profile of these foreign cells was compared with the donor tissue profile, confirming the presence of similar alleles (**Table 1**). Thus, left ovarian tumor analysis showed mixed chimerism, proving the donor origin of this tumor. Furthermore, human leukocyte antigen (HLA) typing was performed on the lung and ovarian lesions. Unique donor HLA was found in both lesions, with ovarian donor HLA A and B identical to donor HLA from the lung lesion, proving donor origin of both lung and ovarian metastases (**Table 1**).

In retrospect, the donor most likely had a low-grade precursor of this vascular malignancy with circulating tumor cells that, in combination with chronic immunosuppression, resulted in late-onset donor-related vascular lung lesions in the recipient after 3 years, followed by liver and ovarian metastases. Eurotransplant, responsible for the allocation of donor organs in this patient, was informed of this serious adverse event. To our knowledge, the other recipients related to this multi-organ donor remained tumor-free: one kidney recipient died from septic shock due to a perianal abscess, the liver recipient died of an unknown cause 2 months post-transplant, and the other kidney recipient is alive and closely monitored after additional investigations were reassuring.

## Discussion

Organ transplantation is a well-established treatment modality for many end-stage diseases but, as with most medical interventions, carries a significant risk of acute or chronic complications. One potential complication is unexpected disease transmission from the donor to the recipient, estimated to occur in less than 1% of transplant procedures (8, 9). Extensive literature on infectious disease transmission is available, but data on cancer transmission are scarce and primarily include case reports and small series. Therefore, an accurate estimate of transmission risk is difficult (3, 4, 10, 11). According to the United Kingdom Transplant Registry, which identified recipients with donor-related malignancy between 2001 and 2010, the incidence was 0.06% (of which 33% renal cell carcinoma and 28% lung carcinoma) (3). This incidence was higher than the 0.01% reported by the Organ Procurement Transplant Network Disease Transmission Advisory Committee/United Network for Organ Sharing in 2002 (4). Overall, the incidence of donor-related malignancy is currently estimated at 0.02%–0.2% among solid organ transplant patients.

After transplantation, malignancies can occur in three ways: patient-related *de novo* occurrence or recurrence of malignancy or donor-related malignancy. Donor-related malignancies mostly occur early after transplantation and can be either due to transmission of occult tumor cells at the time of transplantation (donor-transmitted malignancy) or due to tumor arising in donor-origin cells without prior malignancy in the donor (donor-derived malignancy) (12–15). The latter category may include post-transplant lymphoproliferative disorders that are mostly donor-derived after hematopoietic stem cell transplantation but recipient-derived after solid organ transplantation. Additionally, there is potential for development of tumors due to transmission of oncogenic viruses, such as Epstein–Barr virus, human papillomavirus, human T-lymphotropic virus, or human herpesvirus 8 (12). Our patient was Epstein–Barr virus seropositive at the time of transplantation with multiple negative blood PCRs over the years after transplantation and with no arguments for post-transplant lymphoproliferative disease on histopathology at any time after transplantation. In addition, there were no arguments for Kaposi’s sarcoma with negative staining for human herpesvirus 8.

To prevent donor-transmitted malignancy, careful evaluation of a potential donor is exceedingly important. In the early days of organ transplantation, organs from donors with disseminated malignancies were used, resulting in an unacceptable increase of malignancies in transplant recipients (12, 16). This has led to the avoidance of organs from most donors with a history of malignancy. However, increasing demand for organs resulted in the use of expanded criteria donor organs, with acceptance of older donors, donors with mild disease (e.g., arterial hypertension and diabetes), or donors with smoking history (12). Some patients with a *history* of malignancy may nevertheless be considered potential donors, depending on the type of cancer, interventions given, and interval between diagnosis/cure and organ donation. The same is true for donors with *active* malignancies with a minimal to low risk for transmissions, such as low-grade skin cancer, carcinomas *in situ* of the uterine cervix, and low-grade primary central nervous system tumors (10, 13, 16–21). Donors with a history of adenocarcinoma (except carcinoma *in situ*) are usually avoided, especially if there is a risk of the late appearance of metastasis, such as after breast cancer. Transmission rates vary between different tumor types and even with the type of organs transplanted; risk categorizations for specific malignancy types are available (10, 17). Due to this careful evaluation, tumor transmission is currently an uncommon cause of post-transplant malignancy (13, 17–19). However, cancer transmission has also been reported from donors without a known history of cancer; and with an increasing proportion of older donors, the likelihood of occult cancer may increase (3). Noteworthy, the time window between the prelevation of the donor organ and transplantation into the recipient is limited due to ischemia time and does not allow extensive donor histological or tissue genetic analysis.

The herein-reported case is unique due to the late onset and very slow time course of development of vascular tumors over a period of 4 years, together with state-of-the-art TNGS and HLA typing to confirm donor origin. It is difficult to know the exact origin of the tumor, but our findings suggest that the vascular lesion of the donor might have been a precursor tumor and that chronic immunosuppression may have contributed to the occurrence of disseminated vascular malignancy. In that respect, our patient differed from the other recipients of this donor, namely, the dose and combination of immunosuppressive agents are lower in liver and kidney transplants. Lung transplant recipients are generally treated with a triple immunosuppressive regimen, consisting of a calcineurin inhibitor, a cell cycle inhibitor, and corticosteroids, because of a high incidence of acute rejection and the fact that chronic lung allograft dysfunction remains the major factor limiting long-term survival (22).

Even if morphological findings are identical, host-related and donor-related malignancies are distinct diseases, with potentially different treatments (which may implicate organ explantation if possible), and may impact other organ recipients from the same donor, hence the importance of establishing the exact genome-of-origin; a sensitive method using multiple STR loci or single-nucleotide polymorphisms is recommended in gender-identical transplants, whereby distinction can be made in almost all cases (12).

## Conclusion

We describe a unique case of donor-transmitted angiosarcoma, with late post-transplant presentation as non-specific vascular tumors in the lungs and liver for up to 6 years after transplantation, yet with the evolution toward a Krukenberg tumor and finally disseminated lethal disease. Donor origin of tumors detected in solid organ transplant recipients should always be suspected in case of an uncommon disease course or histology, and proper tissue-based diagnosis should be pursued, given the possible implications for treatment and outcome of other organ recipients from the same multi-organ donor.

## The Leuven Lung Transplant Group

This includes the following important collaborators of our lung transplant program who were directly involved in the care of our lung transplant recipients during the past years:

Jonas Yserbyt, PhD

Arne P. Neyrinck, PhD

Veronique Schaevers, MSc

Bruno Desschans, MSc

Dirk Claes, MSc

Karen Denaux, MSc

Paul De Leyn, PhD

Herbert Decaluwé, PhD

Hans Van Veer, MD

Lieven Depypere, PhD

Marie-Paule Emonds, PhD

## Data Availability Statement

The original contributions presented in the study are included in the article/**Supplementary Materials**, further inquiries can be directed to the corresponding author.

## Ethics Statement

The studies involving human participants were reviewed and approved by the Ethics Committee of the University Hospital of Leuven under agreement S51577. The patients/participants provided their written informed consent to participate in this study. Written informed consent was obtained from the individual(s) for the publication of any potentially identifiable images or data included in this article.

## Author Contributions

SB: performed the research/study, collected the data, and wrote the manuscript. LD: performed the HLA analysis and critically revised the manuscript. LM, IV, and SV: performed genetic analyses and critically revised the manuscript. FJ and PS: the treating oncologists and critically revised the manuscript. BW, RS, and SD: performed the histological analyses and critically revised the manuscript. DV: the treating lung transplant surgeon and critically revised the manuscript. LC: the assisting lung transplant surgeon and critically revised the manuscript. LG, LJD, and GV: the treating lung transplant physicians and critically revised the manuscript. RV: the delegating lung transplant physician over time, coordinated and designed the research/study, collected the data, and critically revised the manuscript. All authors contributed to the article and approved the submitted version.

## Funding

The following authors are supported by a research fellowship but received no specific funding for the current study: RV is a senior clinical research fellow of the Fund for Scientific Research Flanders (FWO). GV and DV are supported by the Broere Charitable Foundation. LC is supported by a KU Leuven University Chair funded by Medtronic.

## Author Disclaimer

The authors confirm that the work described has not been published previously, that it is not under consideration for publication elsewhere, that its publication is approved by all authors and tacitly or explicitly by the responsible authorities where the work was carried out, and that, if accepted, it will not be published elsewhere in the same form in English or in any other language, without the written consent of the copyright holder.

## Conflict of Interest

The authors declare that the research was conducted in the absence of any commercial or financial relationships that could be construed as a potential conflict of interest.

## Publisher’s Note

All claims expressed in this article are solely those of the authors and do not necessarily represent those of their affiliated organizations, or those of the publisher, the editors and the reviewers. Any product that may be evaluated in this article, or claim that may be made by its manufacturer, is not guaranteed or endorsed by the publisher.

## Abbreviations

^18^F-FDG PET-CT, ^18^F-fluorodeoxyglucose PET-CT; HLA, human leukocyte antigen; STR, short tandem repeat; TNGS, targeted next-generation sequencing.
