# Supplementary material for: Case Report: An Unusual Course of Angiosarcoma After Lung Transplantation
Source: Front Immunol. 2022 Jan 3;12:789851. doi: 10.3389/fimmu.2021.789851 (PMC8761760; doi:10.3389/fimmu.2021.789851)

Supplemental Appendix: radiology images in large format

March 2013

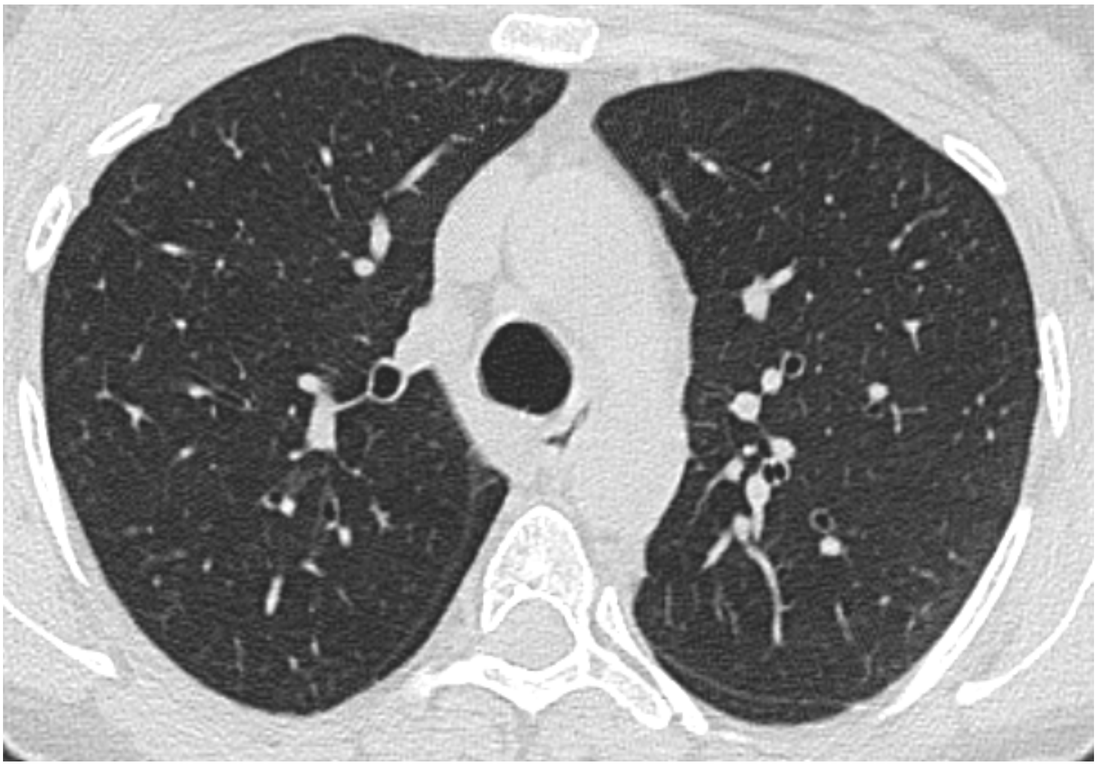

March 2016

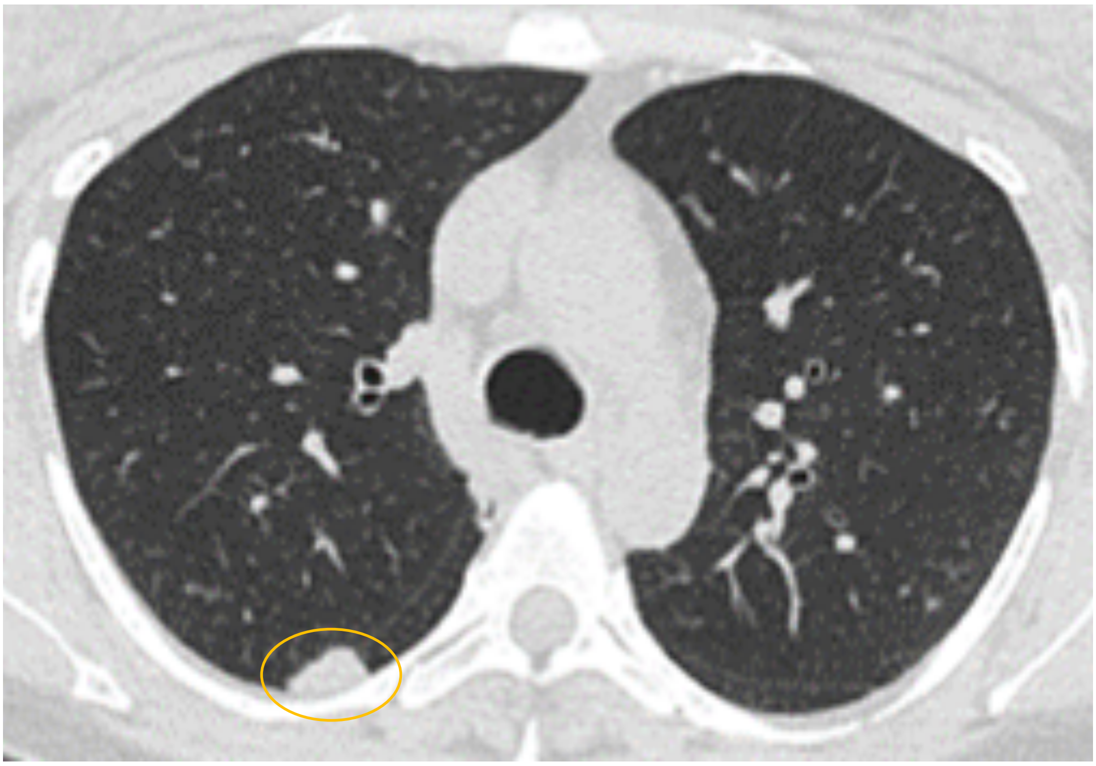

September 2016

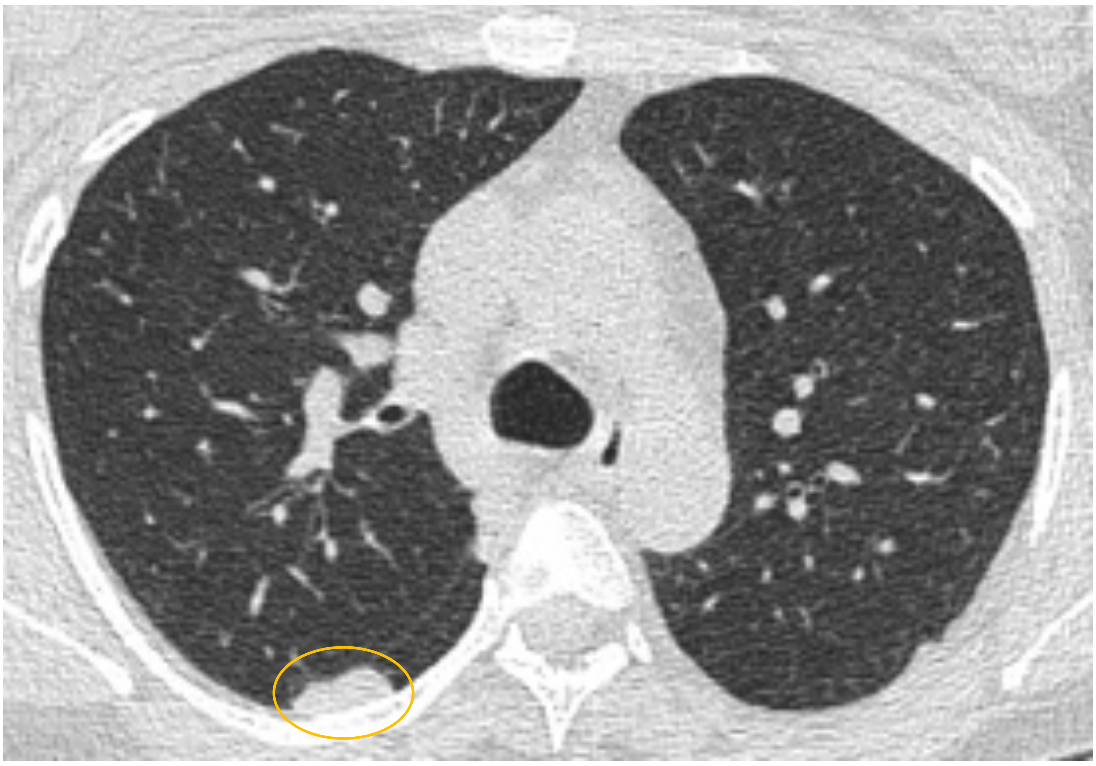

April 2017

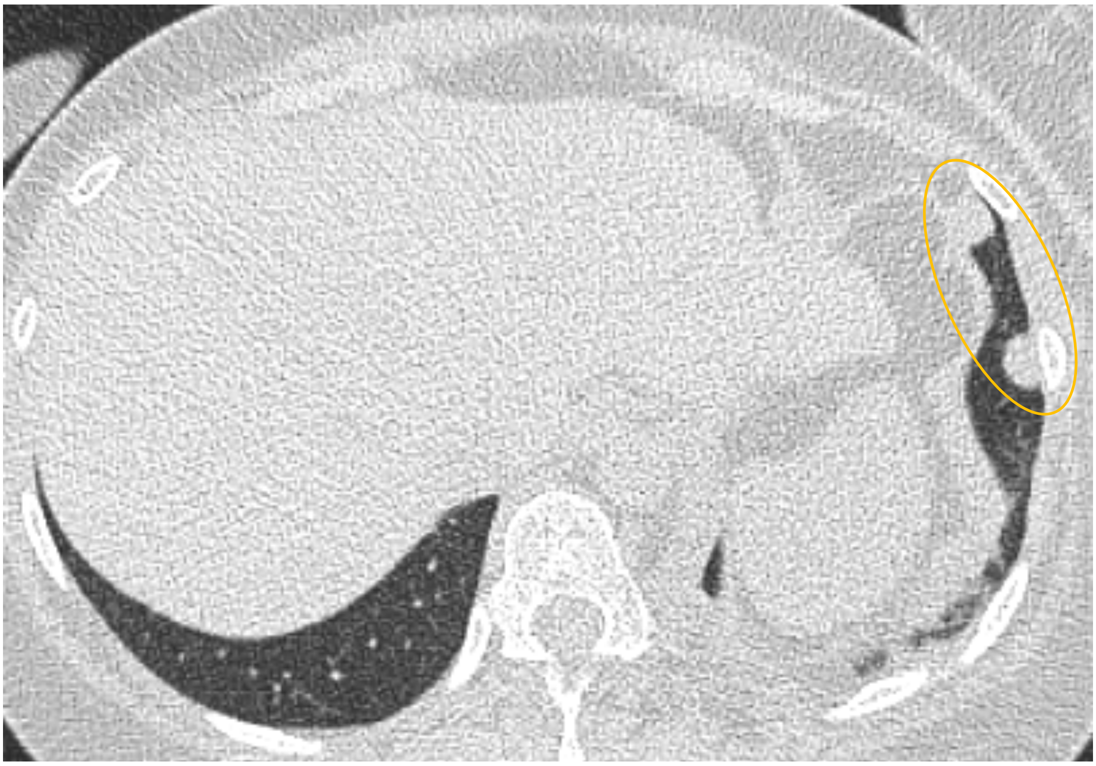

April 2018

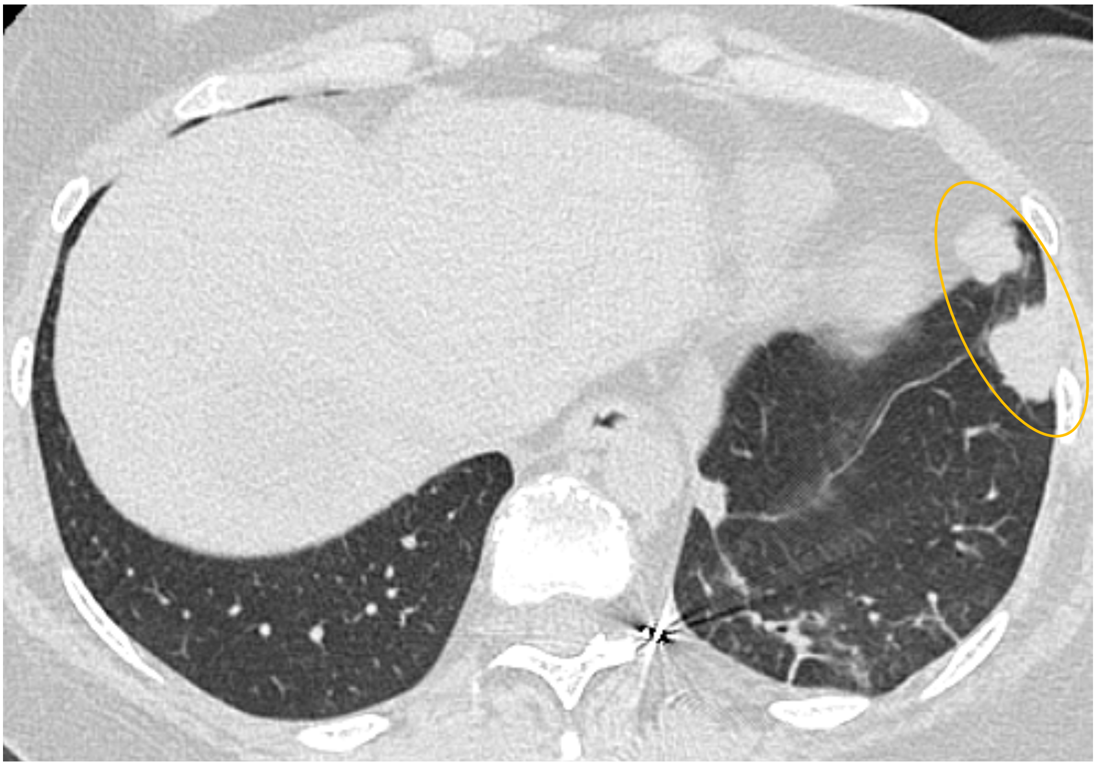

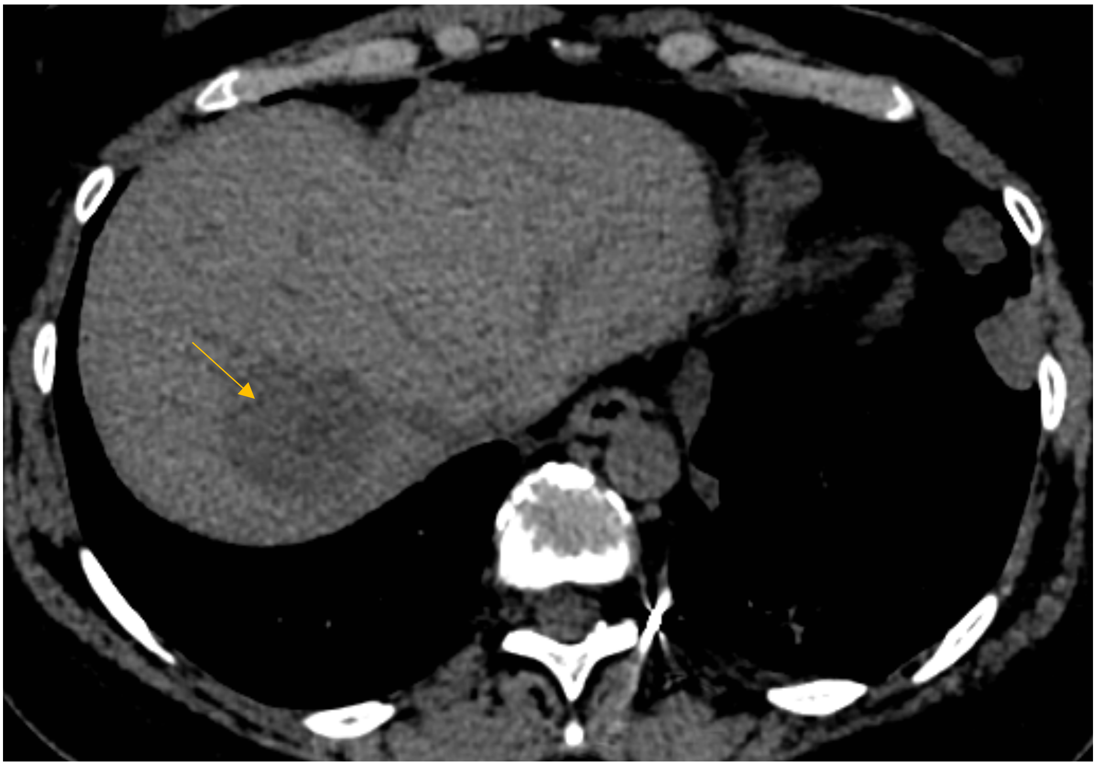

August 2018

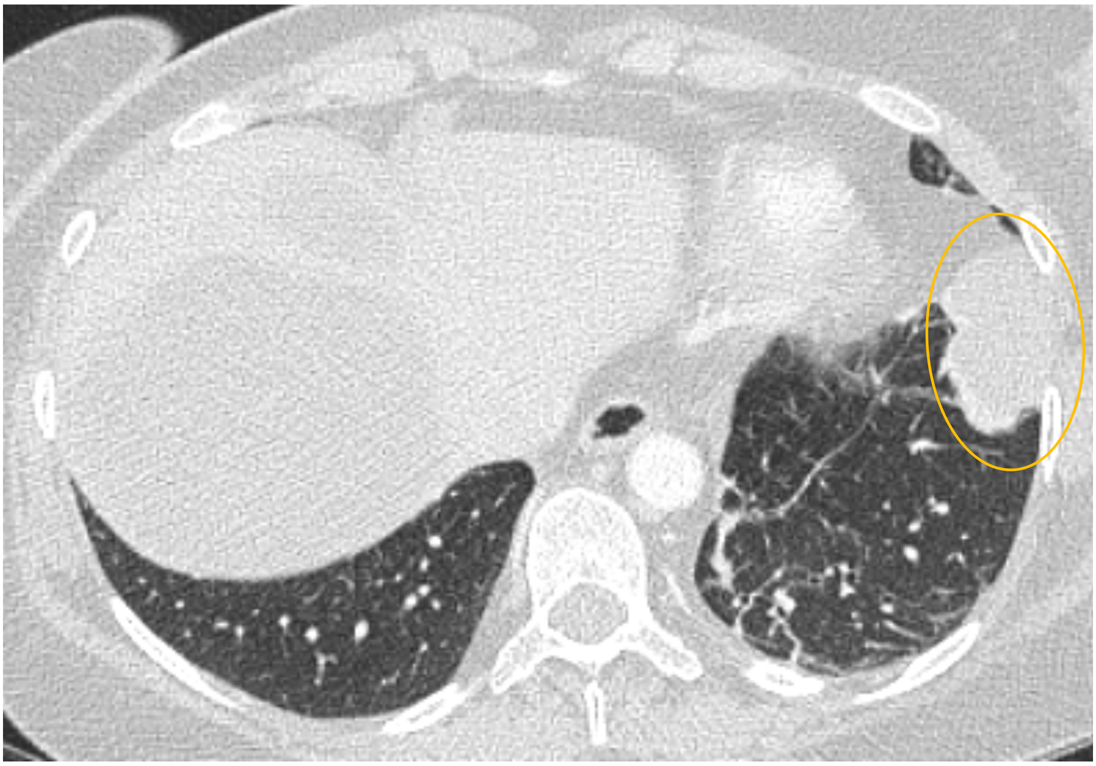

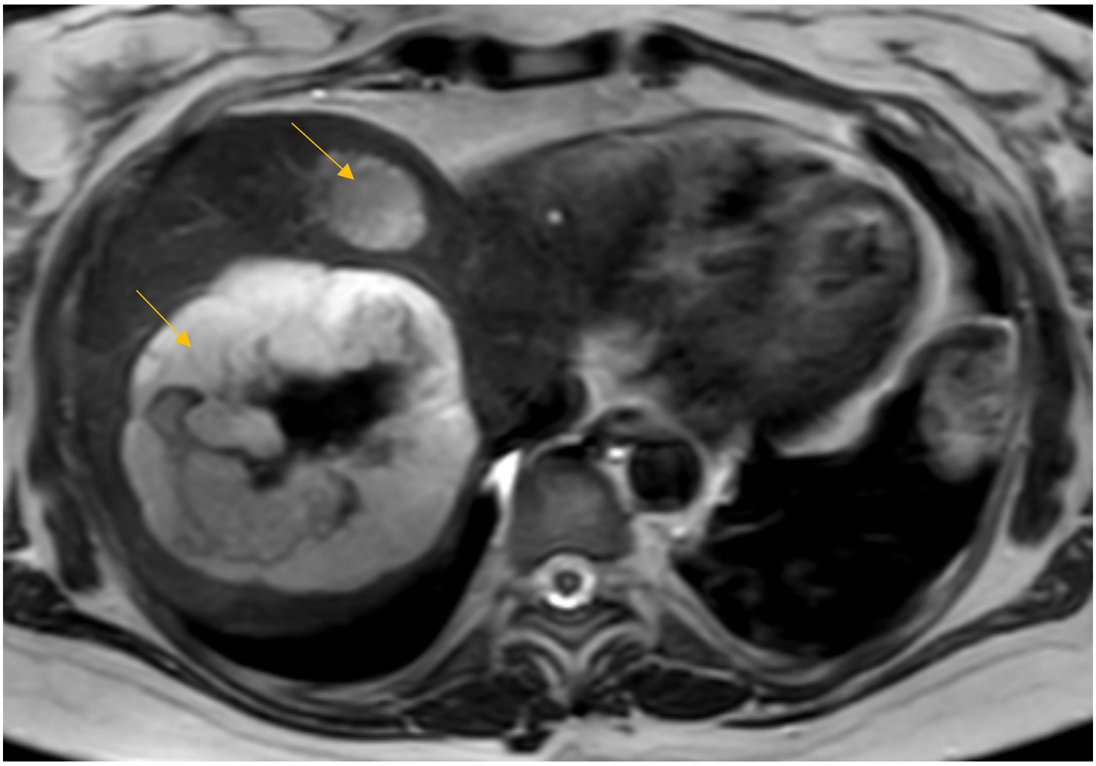

May 2019

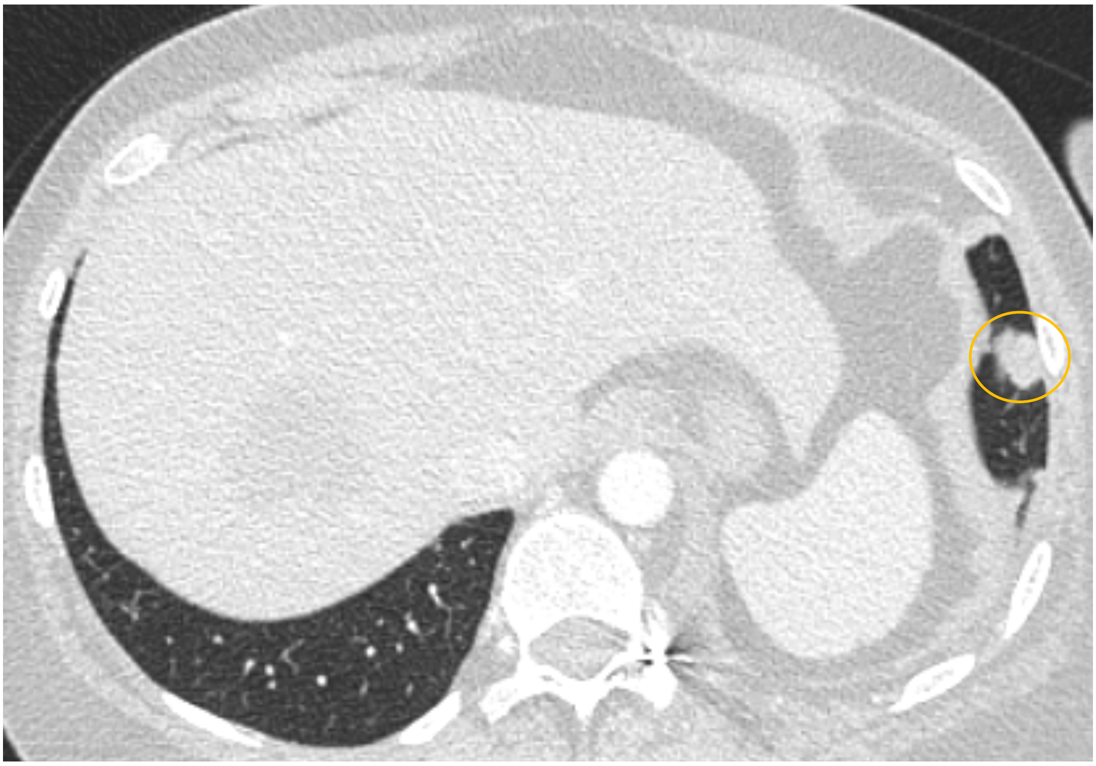

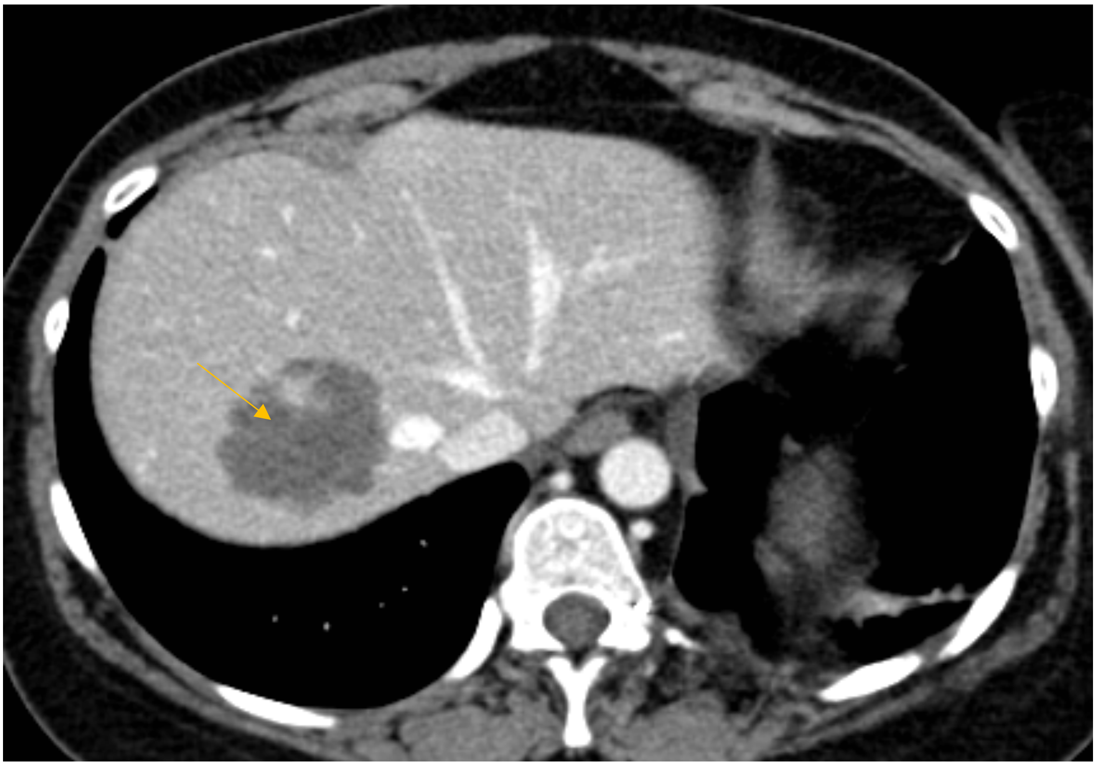

November 2019

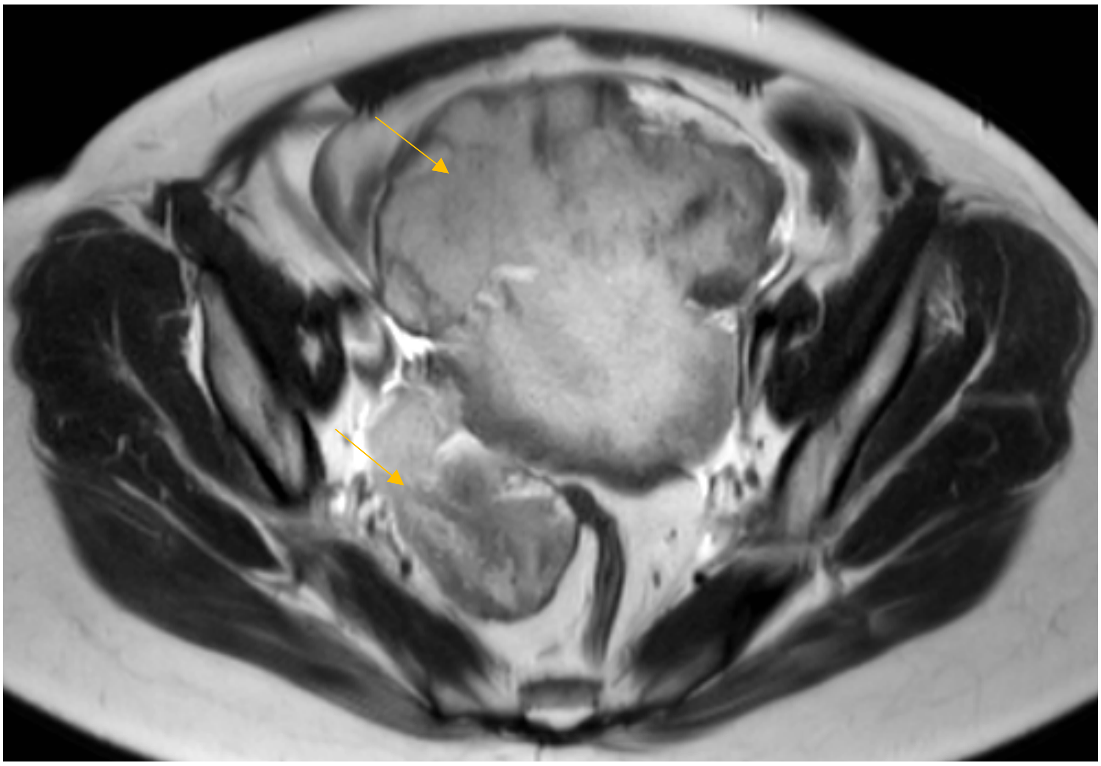

January 2020

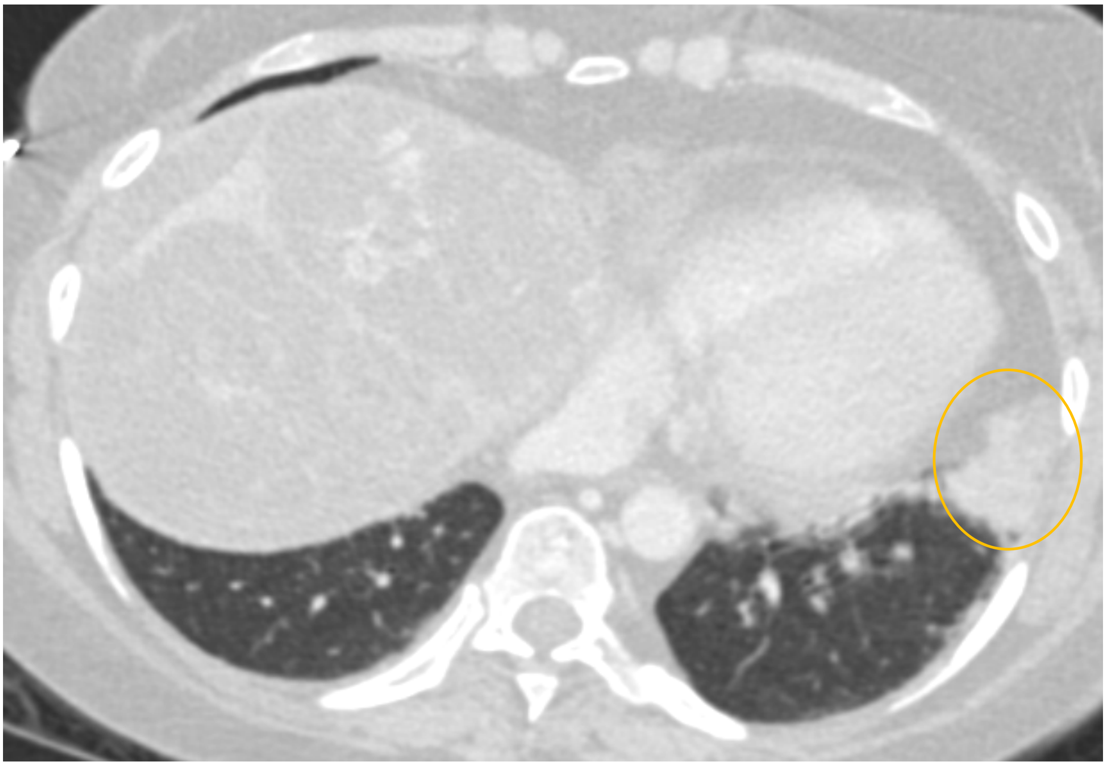

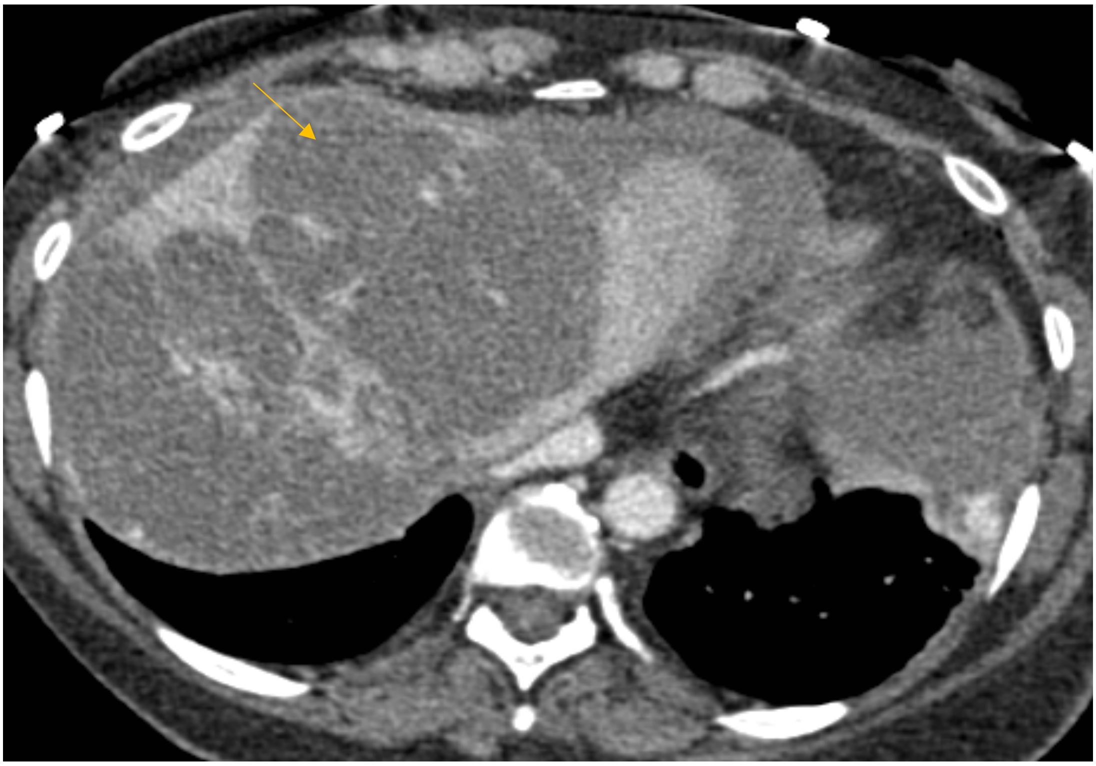

October 2020

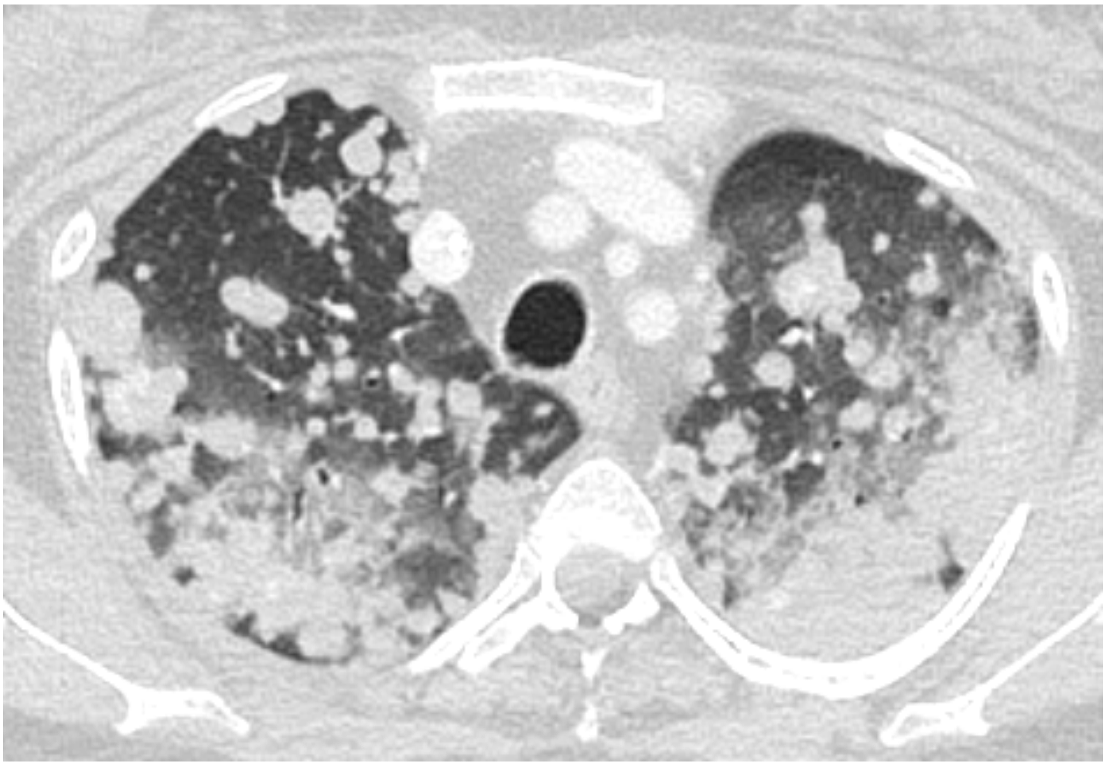

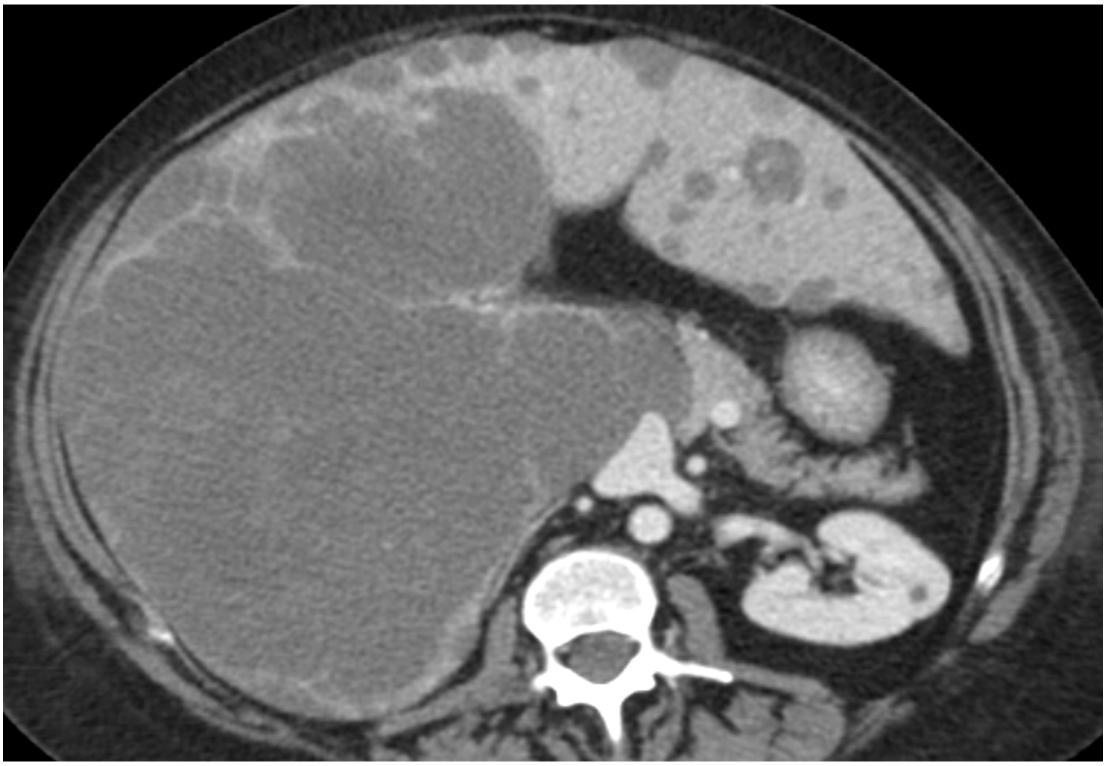

Supplement: Supplementary file 1 [file DataSheet_1.pdf]
